# The Effect of Time Window Length on EEG-Based Emotion Recognition

**DOI:** 10.3390/s22134939

**Published:** 2022-06-30

**Authors:** Delin Ouyang, Yufei Yuan, Guofa Li, Zizheng Guo

**Affiliations:** 1 Institute of Human Factors and Ergonomics, College of Mechatronics and Control Engineering, Shenzhen University, Shenzhen 518060, China; ouyangdelin2021@email.szu.edu.cn (D.O.); yuanyufei2021@email.szu.edu.cn (Y.Y.); 2 National Engineering Laboratory of Integrated Transportation Big Data Application Technology, National United Engineering Laboratory of Integrated and Intelligent Transportation, and Comprehensive Transportation Key Laboratory of Sichuan Province, School of Transportation and Logistics, Southwest Jiaotong University, Chengdu 611756, China; guozizheng@swjtu.edu.cn

**Keywords:** brain–computer interaction, emotion recognition, time window length, electroencephalogram (EEG), experiment-level batch normalization

## Abstract

Various lengths of time window have been used in feature extraction for electroencephalogram (EEG) signal processing in previous studies. However, the effect of time window length on feature extraction for the downstream tasks such as emotion recognition has not been well examined. To this end, we investigate the effect of different time window (TW) lengths on human emotion recognition to find the optimal TW length for extracting electroencephalogram (EEG) emotion signals. Both power spectral density (PSD) features and differential entropy (DE) features are used to evaluate the effectiveness of different TW lengths based on the SJTU emotion EEG dataset (SEED). Different lengths of TW are then processed with an EEG feature-processing approach, namely experiment-level batch normalization (ELBN). The processed features are used to perform emotion recognition tasks in the six classifiers, the results of which are then compared with the results without ELBN. The recognition accuracies indicate that a 2-s TW length has the best performance on emotion recognition and is the most suitable to be used in EEG feature extraction for emotion recognition. The deployment of ELBN in the 2-s TW can further improve the emotion recognition performances by 21.63% and 5.04% when using an SVM based on PSD and DE features, respectively. These results provide a solid reference for the selection of TW length in analyzing EEG signals for applications in intelligent systems.

## 1. Introduction

Human emotion recognition is a critical research topic in brain–computer interaction (BCI) [1,2,3,4]. Most of the current human emotion recognition approaches are based on facial expression images from cameras [5]. However, the real emotion may be hidden behind facial expressions consciously or unconsciously, which would make the camera-based approaches invalid. Moreover, the effectiveness of these approaches would also be limited in poor environments with low illumination or rapidly changing light distributions on human faces (e.g., in nighttime driving). Therefore, using physiological signals to directly recognize human emotions without environmental effects or fake facial expressions is essential.

Among the various physiological signals, electroencephalogram (EEG) has been frequently reported to be closely and directly related with human emotions in previous studies [6,7,8,9,10]. George et al. [11] applied fast Fourier transformation (FFT) and frequency bandpass to extract features from EEG signals and performed emotion recognition in valence and arousal dimensions with a support vector machine (SVM). Asghar et al. [12] proposed a bag of deep features (BoDF) model to reduce the EEG feature dimensionality and adopted an SVM and k-nearest neighbors (KNN) to perform EEG-based emotion recognition. Pan et al. [13] applied the logistic regression (LR) algorithm with Gaussian kernel and Laplacian prior for EEG-based emotion recognition by comparing with Gaussian naive bayes (GNB) and SVM classifiers.

However, most of these studies use the complete EEG samples with different time durations as inputs for model training. This treatment would make the trained model not suitable to be applied for online or real-time emotion recognition because the learned knowledge of the trained model is all about the characteristics of the complete samples for post-detection which is not suitable for online or real-time applications [14]. Moreover, mixing the samples with different durations together would also make the trained model not aware of the changing characteristics of EEG signals in temporal sequences [15,16].

To this end, various time windows (TWs) have been developed and used in signal analysis of EEG temporal sequences. Abtahi et al. [17] used a 20-millisecond TW with 10-millisecond offset to cut EEG signals into a time sequence to determine the suitable analysis model for EEG signals. Lin et al. [18] applied a 1-s TW without overlapping on channels of the EEG data to compute the EEG spectrogram which would be used to investigate the relationship between emotional states and brain activities with machine-learning algorithms. Zheng et al. [19] used a non-overlapped 4-s TW with short-time Fourier transform for extracting EEG features to perform emotion recognition tasks with feature level fusion strategy and decision level fusion strategy, respectively. Zhuang et al. [20] took EEG data in every 5-s TW as material for empirical mode decomposition (EMD), which was beneficial for EEG-based emotion recognition performance. It can be observed that various TW lengths were used in the signal processing of EEG signals. However, there is currently no criterion or prior knowledge on the temporal scale (i.e., TW length) to measure EEG data for emotion recognition.

Moreover, in the development of emotion recognition with EEG data, a significant problem is the negative influence of individual differences [15,16] which leads to diversified EEG response patterns to affect the generalization capabilities of classifiers across subjects. Gianotti et al. [21] reported the neural signatures underlying individual participants when they were being looked at. Matthews et al. [22] found that differences existed in EEG signal responses through an experiment on 150 patients who were asked to perform two signal detection tasks in a complex and simulated operational environment. Meanwhile, many other studies have proposed various data processing methods for subject-independent emotion recognition [15,23,24], but they process EEG data without considering differences among experimental groups.

Therefore, this paper aims to examine the effectiveness of different TW lengths on emotion recognition based on batch normalized EEG signals for better individualized emotion recognition performance. The main contributions of this paper are two-fold. Firstly, the best TW length to extract features for EEG-based emotion recognition is determined. This would fill the research gap on the selection of TW for feature extraction to facilitate EEG-based emotion recognition. Secondly, an experiment-level batch normalization (ELBN) method is newly applied in EEG feature processing to alleviate the negative impact caused by individual differences in different experiments. This method can help extract useful features without being greatly affected by human behavioral differences across experiments, which would greatly improve emotion recognition performances in online or real-time applications.

## 2. Related Work

### 2.1. Characteristics of EEG Signals

EEG signals are non-stationary, non-linear, and non-Gaussian [25]. Recorded as time sequences from multi-channels, EEG signals have high spatial-temporal complexity especially when an enormous amount of EEG data is recorded [26]. In addition, the characteristics of EEG signals related to some specific events will change over time, making EEG-based recognition more difficult and requiring calibration before application [27]. EEG signals also have a relatively low signal-to-noise ratio (SNR) and are susceptible to distortion from artificial interference (e.g., eye movement) [28]. To remove these artifacts and make EEG signals more correlated to the target events, EEG signals are usually calculated in five frequency bands, i.e., delta (1–3 Hz), theta (4–7 Hz), alpha (8–13 Hz), beta (14–30 Hz), and gamma (31–50 Hz) [27]. Useful features can be extracted from these five frequency bands of EEG signals for detailed analysis on specific tasks [29,30].

Another significant characteristic of EEG signals is that EEG is highly subject to various individual differences, which brings difficulties when the pre-trained model is applied on a new subject directly. To address this problem, previous studies have proposed a variety of approaches for EEG data processing. Li et al. [23] proposed a normalization method where EEG signals in each electrode channel of each person was normalized into the range of [0, 1]. Another study [15] presented a domain adaptation method where task-invariant features and task-specific features integrated in unified framework models were adopted to eliminate individual differences. Lu et al. [24] also developed a dynamic entropy-based pattern for subject-independent emotion recognition. However, most of these previous studies take EEG data from different subjects and different experimental batches as an integral whole without considering the influence of both inter-subjects and inter-experiments difference, meaning subject-independent emotion recognition still faces challenges. To further diminish the effect of individual difference, the inter-subjects and inter-experiments differences should be taken into consideration when processing EEG data.

### 2.2. EEG Features

To analyze EEG signals from different perspectives, various features have been extracted to describe brain activity information. Unde et al. [31] used power spectral density (PSD), which was defined as the distribution of signal power over frequency, to show the strength of energy in a frequency domain. Shi et al. [32] applied differential entropy (DE), which was obtained by calculating the entropy of a continuous EEG sequence, to measure the complexity of EEG signals. Frantzidis et al. [33] used amplitude and latency of event-related potentials (ERPs) as features in their research. However, detecting emotion-related ERPs is difficult in online applications as the onset is usually unknown. Kroupi et al. [34] employed the non-stationary index (NSI) to measure the inconsistency of EEG signals, which is defined as the standard deviation of all the means from the EEG signal pieces. Petrantonakis et al. [35] introduced higher order crossings (HOC)-based features to capture the oscillatory pattern of EEG signals [36].

Among these various EEG features, PSD and DE are two commonly used and well-accepted features used to analyze human’s EEG activities. PSD features are used to represent the distribution and energy strength of signal power over a frequency [8,9]. DE are efficient numerical features employed to measure the signal complexity in EEG analysis [37], and they perform well in differentiating EEG signals between low and high frequency energies [7]. It has been demonstrated that PSD and DE features could effectively describe EEG signals to achieve high accuracies for emotion recognition [37]. Therefore, we used PSD and DE features for emotion recognition in this study.

## 3. Dataset and Experiments

The SJTU emotion EEG dataset (SEED) [29] is a popular publicly available EEG dataset for various purposes on emotional analysis. The data collection work was performed by the BCMI laboratory in Shanghai Jiao Tong University in 2015. The ESI NeuroScan system was used to record the EEG data with a sample rate of 1000 Hz. There was a total of 62 electrode channels according to the international 10–20 system for EEG collection.

Researchers usually elicit specific emotions of subjects by audio or video clips and extract the corresponding EEG data for analysis [17,29,38]. Similarly, film clips were used for emotion induction in SEED. To elicit the different target emotions (i.e., positive, neutral and negative), 15 Chinese film clips were selected following the criterion as follows: (a) the length of the whole experiment should be limited in a reasonable range (e.g., 2–5 min) to avoid fatigue, (b) the content of the films should be easily understood without extra explanation, and (c) only one single target emotion can be elicited through the film content. The film clips were edited so that the selected video content for emotion elicitation could be effective during the approximately 4 minutes’ watching. The selected film clips for emotion induction in our experiments are listed in Table 1. There are five film clips for each emotion type. More details of the dataset and experiments can be found in [39].

**Figure 1:**
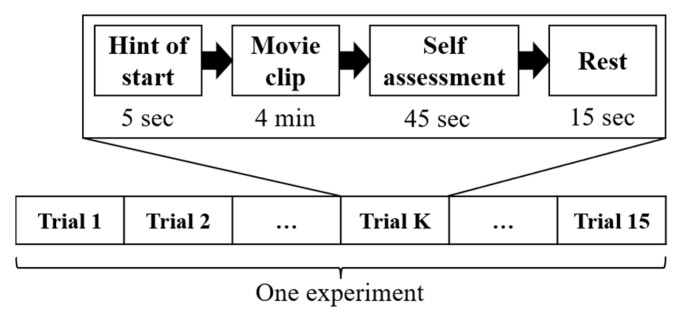
The protocol of the experiments in the SEED dataset [29].

The 15 film clips in Table 1 were separately presented to subjects in 15 trials for one experiment (see Figure 1). In each trial, a starting hint was given 5 s before the start of each clip. When the film clip was finished, 45 s were given for each subject to complete the questionnaire reporting their immediate emotional reactions to the film clip that they had just watched. Details of the questionnaire can be found in [6]. Subsequently, another 15 s were provided for rest before the start of the next trial. The order of emotions presented in the experiments was 1, 0, −1, −1, 0, 1, −1, 0, 1, 1, 0, −1, 0, 1, −1 (1 for positive, 0 for neutral, −1 for negative). According to the presented emotion order, the distribution of the three emotion categories is balanced in each experiment (i.e., each emotion category has 5 corresponding trials in each experiment, and these trials are distributed following the order above). The collected EEG data were downsampled to 200 Hz and filtered with a 0–75 Hz frequency band to filter the noise and remove the artifacts.

There were 15 young subjects (7 males and 8 females; age: 23.27 ± 2.37 years) participating in the experiments in the SEED dataset. Each subject repeated the experiment three times with an interval of one week or longer. Therefore, in the SEED dataset, there were 45 experiments across the 15 subjects. Since each experiment had 15 trails, there were 675 trials in total across the 15 subjects. For each emotion category (i.e., positive, neutral, or negative), there were 225 trials, which means that the emotions were balanced in the SEED dataset.

## 4. Feature Extraction

### 4.1. Power Spectral Density (PSD) and Differential Entropy (DE)

**Figure 2:**
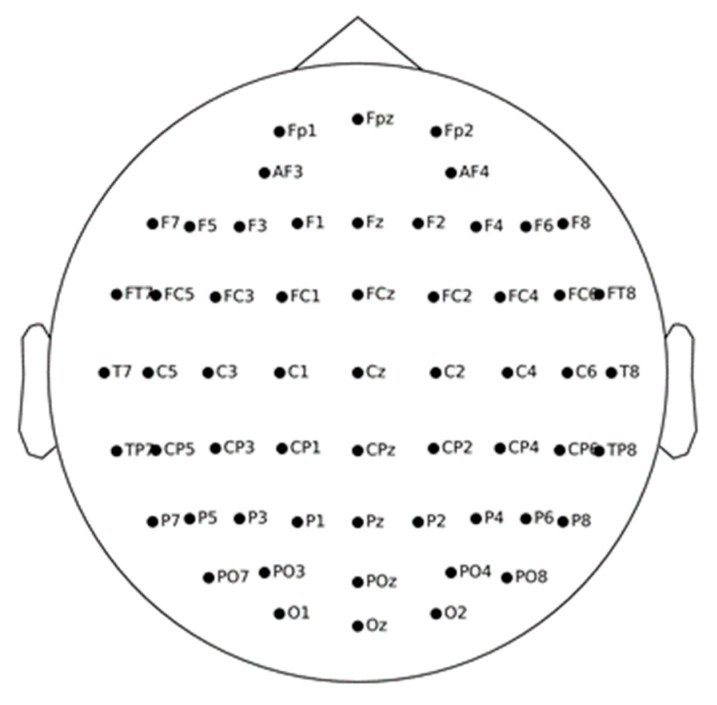
The EEG topo map of the 58 channels.

PSD and DE features [7,37,40] were used to analyze human’s EEG activities in this study. The 64-channel Biosemi Active Two system was applied [7], where 58 channels in the SEED dataset were included with the other 4 channels (PO5, PO6, CB1, CB2) not involved. The positions of the 58 selected channels on the EEG topo map are shown in Figure 2. Each channel was divided into 1-s epochs without overlapping. In each epoch, PSD features and DE features were separately calculated in the delta (1–3 Hz), theta (4–7 Hz), alpha (8–13 Hz), beta (14–30 Hz), and gamma (31–50 Hz) frequency bands, respectively. It has been well reported in previous studies that linear dynamic system (LDS) is a commonly used approach to filter out irrelevant components in EEG data [29,41]. Therefore, LDS was also used in this study.

### 4.2. Extracting Features Based on TW

The minimum duration of the collected trials was about 180 s; hence, each channel of the EEG signals can be segmented into 180 1-s epochs without overlapping. Therefore, there would be 58 × 180 epochs from the collected EEG signals in each trial. PSD features and DE features were calculated in each epoch in the five given frequency bands, respectively. Therefore, the format of features in each trial was defined as (58, 5, 180), where 58 represents 58 channels, 5 represents 5 frequency bands, and 180 represents the total number of features extracted from the epochs in the corresponding trial. In total, there were 675 trials in all the 45 experiments. A total of 11 different lengths of TW were examined to investigate the optimal TW length for EEG data extraction in this study. See Table 2. Both PSD features and DE features were separately calculated and averaged across the epochs in each TW. To compare the effectiveness of the examined features from different TW lengths, six classical classifiers were used for emotion recognition, including KNN, LR, SVM, GNB, Multilayer Perceptron (MLP), and Bootstrap Aggregating (Bagging). These classifiers are the most frequently ones used with high accuracies and strong adaptabilities to different classification tasks [15,42,43]. A machine learning module in Python called sklearn was used to construct models, and the relevant parameter settings are listed in Table 3.

### 4.3. Experimental-Level Batch Normalization (ELBN)

**Figure 3:**
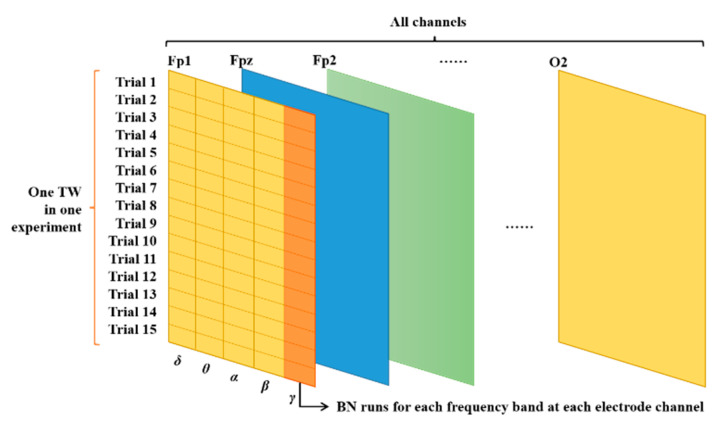
The protocol of ELBN.

To reduce the impact of individual difference on EEG-based emotion recognition [23], an experiment-level batch normalization (ELBN) method was applied on the obtained PSD features and DE features, respectively. The definition of ELBN is shown as follows:

(1) $$F_{BNi}=\frac{F_{i}-F_{min}}{F_{max}-F_{min}}$$

where *F_i_* and *F_BNi_* represent the original value of a specific feature and the value of the feature with ELBN in an experiment, respectively, while *F_min_* and *F_max_* represent the minimum and maximum values of the corresponding feature in the same experiment, respectively. Features extracted from one frequency band in one channel are calculated in each trial, and the features from all 15 trials in the same experiment are normalized following Formula (1). The normalization occurred across 15 trials in one experiment. Each trial can provide one feature from one frequency band in one channel when using a selected TW, and then the 15 trials contribute to the 15 features for normalization. As for the 180 s TW where there is just one TW, the normalization will be performed one time in one experiment with the minimum and maximum values across the 15 trials. Our proposed ELBN is conducted within an experiment to avoid interference or noise from other factors (e.g., change of body status in different experiments), which is newly developed to solve the individual difference and baseline deviation problems in the collected data from different subjects on different days. The protocol of ELBN is shown in Figure 3. Although this idea seems simple, to the best of our knowledge, it has never been used in the previous emotion recognition studies based on EEG signals.

## 5. Results and Discussion

### 5.1. The Effect of TW Length on Emotion Recognition without ELBN

Processed PSD features and DE features with different TW lengths were separately fed into the six classifiers. The training data contain features extracted from 12 trials in each experiment, while the testing data contain features extracted from the other 3 trials from the same experiment. Both training data and testing data are evenly distributed across the three different emotions. A total of 10 random sets (see Table 4) of possible combinations of training data and testing data were selected, and their averaged accuracies in each classifier were used as final recognition results. Although there are some replacement trials in the 10 randomized sets, the distribution of trials for training (or testing) has been randomized as evenly as possible. K-fold is not applied because the order of emotions presented in one experiment across the 15 trials is disordered (i.e., 1, 0, −1, −1, 0, 1, −1, 0, 1, 1, 0, −1, 0, 1, −1 with 1 for positive, 0 for neutral, and −1 for negative). Traditional procedure of k-fold is not suitable for this case when we try to divide 15 trials into a training set and a test set to ensure that the emotions are evenly distributed. If there are no positive, neutral, or negative emotions in the test set, the trained model will not be reliable because of the unbalanced training data, or the testing results for a specific emotion type cannot be computed, making the averaged accuracies not reasonable.

The recognition results of PSD features and DE features when using different TW lengths are shown in Table 5 and Table 6, respectively. The accuracies were calculated using separate EEG frequency bands. The results show that the highest accuracy is achieved when using the LR classifier. The best accuracies when using PSD features or DE features are 67.85% and 78.67%, respectively. The maximum differences across different TW lengths are 3.56% when using PSD features and 2.59% when using DE features. Furthermore, though the number of features increases with the decreasing TW length, the recognition accuracies are barely growing, indicating that the number of features has little influence on recognition results. These results show that the emotion recognition accuracies are limited when using the classical classifiers based on either PSD or DE features, and the accuracy differences when using different TW lengths are not large.

### 5.2. The Effect of TW Length on Emotion Recognition with ELBN

PSD features and DE features with ELBN were separately fed into the six classifiers. Features from the 10 given random sets were used as inputs and their averaged accuracies in each classifier were used as the final recognition results. The recognition results of PSD features and DE features are shown in Table 7 and Table 8, respectively. The results indicate that ELBN performs well on improving emotion recognition accuracies based on EEG features. Compared with the emotion recognition performance when using the same features without ELBN in Table 4 and Table 5, the emotion recognition accuracies when using features with ELBN are greatly improved. The best accuracy of PSD features is up to 79.48% which is 11.53% higher than that without ELBN, and the best accuracy of DE features is up to 82.96% which is 4.29% higher than the number without ELBN. The increased accuracy by ELBN for PSD features is more than 10% for all the examined classifiers, and the greatest improvement (i.e., 21.63%) is achieved when using an SVM. When using DE features, the greatest contribution of our proposed ELBN is 20.67%. These significant accuracy improvements show that our proposed ELBN method can effectively retain more temporal characteristics of the fluctuation trends of EEG signals, contributing to the improvement of emotion recognition based on classical features and classifiers.

When using different TW lengths for emotion recognition, the results shown in Table 6 and Table 7 show that the differences between TW lengths are more obvious than the results in Table 3 and Table 4. The emotion recognition accuracy when using the 2-s or 3-s TW is 6.15% higher than the number when using the 180-s TW for the LR classifier based on PSD features. The highest accuracy when using the 2-s TW based on DE features is 7.18% higher than the accuracy when using the 180-s TW for the LR classifier. By comparing the results from different TW lengths, it can be observed that the best emotion recognition accuracy is achieved when using the 2-s TW together with the LR classifier, hence this TW length together with the LR classifier is used for the following online recognition.

Our results show that the 2-s TW with ELBN has the best emotion recognition performance. In general, a longer length of TW will lead to fewer amounts of input, which is beneficial for reducing computational cost [44], while a shorter length will expand the input scale of features from the temporal dimension, which is capable of capturing EEG transient changes [45]. However, employing a longer length of TW will undermine reading temporal EEG data, while using a shorter length will extend the computing time that is inconvenient to online affective computing. Given that different lengths of TW have different advantages and disadvantages, a suitable TW length that can balance the contradiction between them is required, and our results show that the 2-s TW is an optimal choice with the highest recognition accuracy. As shown in Table 2, compared with the original 1-s TW length, the scale of input features is halved when using the 2-s TW, contributing to decreasing the computational cost. Meanwhile, as shown in Table 5 and Table 6, the 2-s TW is able to keep emotion recognition accuracy at a relatively high level, indicating that temporal characteristics of EEG signals can be effectively extracted for emotion recognition.

ELBN performs well on improving EEG-based emotion recognition according to the results in Table 7 and Table 8. To explore its mechanism for accuracy improvement, samples were randomly selected from the SEED dataset to demonstrate the changes of EEG features after ELBN. Given that differences across EEG-based emotions contribute to emotion recognition [46], significance analysis of PSD features and DE features among the three emotions was conducted on the five frequency bands to explore the sensitivities of features for emotion discrimination before and after applying ELBN. The nonparametric Kruskal–Wallis test was applied on mean values of features in each trial, and the significance analysis results are shown in Table 9. The results show that there are more features with statistical significances among the examined emotions after applying ELBN, indicating that feature sensitivities to human emotions increase after applying ELBN. This would probably be the reason for recognition accuracy improvement after applying ELBN.

### 5.3. Online Emotion Recognition

**Figure 4:**
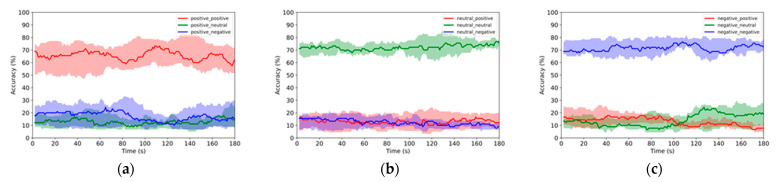
Online emotion recognition results when using PSD features. The legends are described as “ground truth emotion _ online predicted emotion” (e.g., positive _ positive means that the ground truth emotion and online predicted emotion are both positive, while positive _ negative means that the ground truth emotion is positive, but the online predicted emotion is negative.). The solid lines represent the median values of probabilities for online predicted results and the boundaries of the shadow areas illustrate the 25th percentile and 75th percentile values: (**a**) online recognition of positive emotion samples; (**b**) online recognition of neutral emotion samples; (**c**) online recognition of negative emotion samples.

**Figure 5:**
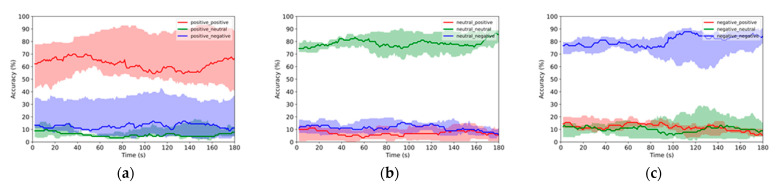
Online emotion recognition results when using DE features: (**a**) online recognition of positive emotion samples; (**b**) online recognition of neutral emotion samples; (**c**) online recognition of negative emotion samples.

A 2-s sliding TW with a 1-s step was used for online emotion recognition with the LR classifier. The 10 random sets were used with 540 × 10 samples for training and 135 × 10 samples for testing, where 540 and 135 are the numbers of samples for training and testing, respectively, in one random set, and the number 10 means that there are 10 random sets. In total, 675 × 10 × 179 TWs (i.e., total samples in one random set × total number of sets × TWs used in one sample) were used to examine the online emotion recognition performance, and the mean accuracy of the testing samples was used as the final output. The results when using PSD features or DE features are illustrated in Figure 4 and Figure 5, respectively. The obvious distance between the red area and blue/green areas in Figure 4a indicate that the positive emotion samples can be correctively recognized without being mistakenly recognized as neural or negative emotion samples. Larger gaps can be found in Figure 4b,c indicating that the neural and negative emotion samples can be more easily recognized without confusing them with the other emotion samples. Similar trends can be found in the results on DE features in Figure 5. These results show that the 2-s TW can be successfully used for online emotion recognition based on EEG signals with ELBN.

As a common sense, human emotion usually lasts much longer than 10 s and can even last several minutes [47]. Using a short TW to continuously recognize human emotion can support online emotion monitoring applications. Given a reasonable range of TW length, a shorter TW is beneficial for a more time-efficient solution for online applications because the recognition would be delayed if a longer TW is used, particularly at the beginning of EEG signal recording where the first recognition result will only be output when the EEG signal sequence with the required time length is collected. However, there is a lack of evidence on the appropriate TW length for EEG-based emotion recognition from a psychological perspective because EEG signals are easily affected by various factors which may differ across individuals [25]. Therefore, it is difficult to determine the best TW length. To simplify this problem, we used recognition accuracy as the evaluation index for TW selection. Even shorter TW was used in previous studies for EEG data processing. For example, Abtahi et al. [17] used EEG data from a 20-millisecond TW to train long-short-term memory (LSTM) and deep belief network (DBN) for emotion analysis.

### 5.4. Influence of TW Length on Emotion Recognition

The selection of TW length mainly affects emotion recognition accuracy as well as model complexity caused by the number of input features. As shown in Table 2, a longer TW corresponds to fewer input features, which means that the model complexity would be lower. However, fewer input features when using a longer TW may lead to a lower recognition accuracy. The SVM classifier results in Table 5 and Table 6 show that the highest recognition accuracy is achieved when the TW length is 30 s and 5 s for PSD and DE features without ELBN, respectively. However, the recognition accuracy reaches a bottleneck and would not continuously increase with the shortening TW length. The results with ELBN in Table 7 and Table 8 show similar trends.

Given that a shorter TW can capture more features with a higher model complexity, but the recognition accuracy would not continuously increase with the shortening TW length, a balanced TW length selection strategy should be considered, which would be another interesting topic for further investigation. Although the LR results in Table 7 and Table 8 show that the highest accuracy is achieved when using the 2-s TW, using a slightly longer TW (e.g., 3-s) would also be a good choice due the generally stable accuracy performance with a lower model complexity. Determining which TW is the best choice would rely on the selection criteria. In this paper, we do not focus on the balance strategy between recognition accuracy and model complexity but just use the recognition accuracy as the selection criteria. In future work, how to reasonably select a TW by comprehensively considering recognition accuracy and model complexity needs deeper analysis.

Another problem is that different frequency bands with different characteristics have different sensitivities to emotions [48], leading to the result that different bands may have different optimal TW lengths. In our experiments, to evaluate the comprehensive performance of TWs, features extracted from different frequency bands are put together for training or testing, making it impossible to investigate the selection of TW length for each frequency band. We will further investigate this interesting topic in our future work.

## 6. Conclusions

The effectiveness of different time window (TW) lengths on emotion recognition is examined based on EEG signals before and after applying experiment-level batch normalization (ELBN). The results show that the highest recognition accuracy is achieved when using the 2-s TW for feature extraction. The highest accuracies when using PSD features and DE features are 79.48% and 82.96%, respectively. The developed ELBN increases the feature sensitivities to emotion discrimination, which greatly contributes to the recognition accuracy improvement. A limitation of this study is that only the classical classifiers are used for emotion recognition. Advanced algorithms based on neural networks and deep learning have been extensively developed for emotion recognition in recent years [48,49,50,51]. Our future work will focus on deploying the explored sensitive features after ELBN in the 2-s sliding TW for emotion recognition using advanced algorithms and analyzing the selection of TW length for each frequency band.

## Figures and Tables

**Table 1:** The selected film clips used to elicit target emotions in the SEED dataset.

No.	Emotion	Film Clip Sources	#Clips
1	negative	Tangshan Earthquake	2
2	negative	Back to 1942	3
3	positive	Lost in Thailand	2
4	positive	Flirting Scholar	1
5	positive	Just Another Pandora’s Box	2
6	neutral	World Heritage in China	5

**Table 2:** The detailed information of TW lengths in each trial. *N* is the number of calculated features in each trial for frequency band of a selected channel.

TW Length (s)	Number of TWs in Each Trial	Epochs Contained in Each TW	Feature Format (58 × 5 × *N*)
180	1	180	58 × 5 × 1
90	2	90	58 × 5 × 2
60	3	60	58 × 5 × 3
30	6	30	58 × 5 × 6
20	9	20	58 × 5 × 9
10	18	10	58 × 5 × 18
5	36	5	58 × 5 × 36
4	45	4	58 × 5 × 45
3	60	3	58 × 5 × 60
2	90	2	58 × 5 × 90
1	180	1	58 × 5 × 180

**Table 3:** Parameter settings of the examined classifiers in sklearn. Those parameters that are not listed in this table are set as the default values, while “\” represents that all the used parameters are the default values.

Classifier	Parameter Setting
KNN	n_neighbors = 5, p = 2, metric = ‘minkowski’
LR	solver = ‘liblinear’, random_state = 10
SVM	random_state = 10
GNB	\
MLP	solver = ‘lbfgs’, alpha = 1e−5, hidden_layer_sizes = (100, 3), random_state = 1, max_iter = 1e5
Bagging	base_estimator = lr, n_estimators = 500, max_samples = 1.0, max_features = 1.0, bootstrap = True, bootstrap_features = False, n_jobs = 1, random_state = 1 (lr = sklearn.linear_model.LogisticRegression(solver = ‘liblinear’, random_state = 1))

**Table 4:** Details of the 10 sets used for training and testing. Please note that the 15 trials are numbered from 0–14.

Set #	The Trials Used for Training	The Trials Used for Testing
1	[0, 1, 2, 4, 5, 6, 9, 10, 11, 12, 13, 14]	[3, 7, 8]
2	[0, 1, 2, 3, 6, 7, 8, 9, 10, 12, 13, 14]	[4, 5, 11]
3	[1, 3, 4, 5, 6, 7, 8, 9, 10, 11, 12, 14]	[0, 2, 12]
4	[0, 1, 3, 4, 5, 6, 7, 8, 9, 10, 11, 14]	[2, 12, 13]
5	[0, 1, 2, 3, 4, 5, 7, 9, 10, 11, 13, 14]	[6, 8, 12]
6	[0, 1, 2, 5, 6, 7, 9, 10, 11, 12, 13, 14]	[3, 4, 8]
7	[0, 1, 2, 3, 4, 6, 7, 8, 9, 11, 12, 13]	[5, 10, 14]
8	[0, 2, 3, 4, 5, 7, 9, 10, 11, 12, 13, 14]	[1, 6, 8]
9	[0, 3, 4, 5, 6, 7, 8, 10, 11, 12, 13, 14]	[1, 2, 9]
10	[0, 1, 2, 3, 4, 5, 6, 8, 10, 11, 12, 13]	[7, 9, 14]

**Table 5:** The recognition results (mean accuracy and standard deviation) of PSD features extracted from separate frequency bands when using different classifiers under different TW lengths without ELBN. The highest and second-highest accuracies of each classifier are highlighted in bold red and bold blue, respectively.

TW Length (s)	KNN	LR	SVM	GNB	MLP	Bagging
180	54.22(3.18)	66.81(5.81)	49.48(6.32)	39.63(5.42)	62.59(5.52)	66.30(5.72)
90	55.33(2.64)	**67.85(6.05)**	51.78(4.96)	**39.85(6.36)**	**67.78(4.77)**	68.07(6.37)
60	**56.37(2.43)**	**67.70(6.13)**	**52.89(4.19)**	**39.70(6.84)**	66.67(5.17)	**68.67(5.76)**
30	**56.67(3.24)**	67.41(7.53)	**53.04(4.21)**	39.56(6.75)	**66.81(5.77)**	68.15(6.37)
20	**56.37(2.78)**	67.63(6.50)	**52.89(4.01)**	39.56(6.94)	66.52(4.63)	67.78(5.51)
10	56.15(2.77)	66.37(6.34)	52.74(4.08)	39.41(6.99)	63.63(5.32)	**68.30(5.61)**
5	56.07(2.86)	65.70(6.12)	52.81(4.01)	39.48(6.98)	65.26(4.99)	67.63(5.40)
4	56.00(2.84)	65.41(6.10)	52.81(4.01)	39.56(6.98)	66.22(4.99)	67.26(5.28)
3	56.00(2.84)	65.63(5.93)	52.81(4.01)	39.56(6.98)	64.96(5.10)	67.19(5.52)
2	56.00(2.84)	65.63(5.15)	52.81(4.01)	39.56(6.98)	61.85(7.59)	66.96(5.76)
1	56.00(2.84)	65.78(4.70)	52.81(4.01)	39.56(6.98)	65.04(4.34)	67.19(6.04)

**Table 6:** The recognition results (mean accuracy and standard deviation) of DE features extracted from separate frequency bands when using different classifiers under different TW lengths without ELBN. The highest and second-highest accuracies of each classifier are highlighted in bold red and bold blue, respectively.

TW Length (s)	KNN	LR	SVM	GNB	MLP	Bagging
180	65.78(4.81)	77.19(7.45)	70.00(4.09)	48.30(2.91)	66.30(8.91)	76.74(6.35)
90	**66.59(5.53)**	76.81(7.62)	71.48(5.90)	48.96(2.93)	72.67(5.35)	77.41(7.11)
60	**66.37(5.21)**	76.22(7.65)	71.56(6.23)	48.96(2.82)	**76.15(6.97)**	77.70(7.11)
30	66.22(5.06)	76.81(7.25)	71.93(6.21)	48.96(2.87)	74.81(6.76)	78.00(6.40)
20	65.85(5.04)	**78.67(6.61)**	72.07(6.25)	48.96(2.87)	**75.70(6.16)**	78.30(6.52)
10	66.15(4.91)	**78.67(5.37)**	**72.22(6.44)**	**49.19(3.03)**	72.07(8.17)	78.30(5.73)
5	66.30(4.85)	**78.59(5.21)**	**72.59(6.56)**	**49.26(3.24)**	73.48(7.52)	78.52(5.59)
4	66.30(4.85)	78.30(5.41)	**72.59(6.56)**	**49.26(3.24)**	74.67(5.79)	78.74(5.72)
3	66.30(4.85)	78.15(5.04)	**72.59(6.56)**	**49.26(3.24)**	75.11(8.21)	78.74(6.09)
2	66.30(4.85)	77.70(5.08)	**72.59(6.56)**	**49.26(3.24)**	68.59(5.12)	**78.81(6.19)**
1	66.30(4.85)	77.19(5.69)	**72.59(6.56)**	**49.26(3.24)**	73.11(7.96)	**78.89(6.15)**

**Table 7:** The recognition results (mean accuracy and standard deviation) of PSD features extracted from separate frequency bands when using different classifiers under different TW lengths with ELBN. The highest and second-highest accuracies of each classifier are highlighted in bold red and bold blue, respectively.

TW Length (s)	KNN	LR	SVM	GNB	MLP	Bagging
180	64.89(4.06)	73.33(6.87)	72.59(3.94)	56.44(5.94)	72.37(4.76)	74.37(5.55)
90	65.85(3.80)	77.93(6.56)	73.41(3.86)	59.78(6.01)	73.04(4.58)	78.37(6.56)
60	67.48(3.55)	77.04(6.93)	73.70(4.64)	**60.44(5.95)**	**74.30(8.73)**	77.78(6.20)
30	67.56(2.94)	77.48(5.94)	**74.37(4.94)**	60.37(6.01)	71.26(7.97)	77.78(5.26)
20	67.26(3.06)	77.85(6.09)	**74.37(4.97)**	**60.44(6.30)**	**74.30(5.14)**	78.67(5.13)
10	67.63(3.16)	79.04(6.03)	**74.44(5.18)**	60.30(6.02)	69.93(5.19)	78.67(5.52)
5	67.70(3.11)	79.04(6.29)	**74.44(5.13)**	**60.37(6.13)**	73.41(6.56)	**79.41(5.79)**
4	**67.78(3.07)**	79.04(6.17)	**74.44(5.13)**	**60.37(6.13)**	73.26(4.25)	79.33(5.76)
3	**67.78(3.07)**	**79.48(5.82)**	**74.44(5.13)**	**60.37(6.13)**	72.96(6.96)	**79.41(5.56)**
2	**67.85(3.03)**	**79.48(5.81)**	**74.44(5.13)**	**60.37(6.13)**	70.59(7.61)	79.33(5.51)
1	**67.85(3.01)**	**79.11(5.72)**	**74.44(5.13)**	**60.37(6.13)**	**73.85(6.53)**	**79.56(5.30)**

**Table 8:** The recognition results (mean accuracy and standard deviation) of DE features extracted from separate frequency bands when using different classifiers under different TW lengths with ELBN. The highest and second-highest accuracies of each classifier are highlighted in bold red and bold blue, respectively.

TW Length (s)	KNN	LR	SVM	GNB	MLP	Bagging
180	70.67(5.63)	75.78(8.55)	75.70(5.47)	67.48(6.22)	73.33(7.50)	77.70(7.21)
90	**74.15(4.91)**	79.70(8.02)	77.41(6.01)	69.26(6.98)	75.70(6.82)	80.89(7.79)
60	72.37(5.93)	79.04(8.29)	**77.78(5.80)**	69.41(6.84)	75.78(7.16)	80.22(7.65)
30	73.19(5.47)	81.56(7.10)	77.26(4.94)	69.41(6.98)	73.70(7.28)	81.48(7.37)
20	72.96(5.31)	81.56(7.74)	77.48(5.32)	**69.63(6.94)**	76.15(7.84)	81.26(7.73)
10	73.26(5.41)	82.37(6.50)	77.56(5.13)	**69.48(6.89)**	76.22(6.72)	82.15(6.64)
5	73.41(5.31)	82.59(6.34)	77.56(5.13)	69.41(6.74)	**78.37(7.47)**	**82.30(6.67)**
4	73.41(5.22)	82.44(6.60)	**77.63(5.24)**	69.41(6.74)	77.04(7.40)	**82.37(6.57)**
3	73.41(5.22)	**82.81(6.82)**	**77.63(5.24)**	69.41(6.74)	76.00(6.50)	**82.30(6.42)**
2	**73.48(5.22)**	**82.96(6.94)**	**77.63(5.24)**	69.41(6.74)	**77.19(6.76)**	82.22(6.55)
1	73.33(5.16)	82.52(7.13)	**77.63(5.24)**	69.41(6.74)	76.30(6.44)	82.15(6.81)

**Table 9:** The recognition results (mean accuracy and standard deviation) of DE features with different TW lengths. The red color highlights the results with statistical significance (*p* ≤ 0.05).

Feature	Frequency Band	Processing	Fp1	Fpz	Fp2	AF3	AF4	C3	C1	Cz	C2	C4	C6	P1	Pz	P2	P4	P8	PO7	PO3
PSD	Delta	Without ELBN	.000	.000	.000	.000	.000	.003	.019	.712	.066	.002	.000	.301	.086	.109	.085	.000	.036	.156
	Theta		.017	.022	.030	.029	.325	.024	.235	.798	.513	.129	.001	.036	.008	.019	.005	.000	.001	.013
	Alpha		.582	.632	.671	.367	.767	.334	.482	.994	.673	.623	.172	.484	.439	.484	.449	.046	.149	.406
	Beta		.437	.252	.497	.398	.539	.000	.020	.814	.082	.001	.000	.326	.216	.368	.092	.000	.000	.001
	Gamma		.218	.032	.246	.064	.457	.000	.000	.454	.002	.000	.000	.000	.000	.000	.000	.000	.000	.000
	Delta	With ELBN	.000	.000	.000	.000	.000	.000	.000	.038	.000	.000	.000	.000	.000	.000	.000	.000	.000	.000
	Theta		.000	.000	.000	.000	.000	.000	.000	.001	.000	.000	.000	.000	.000	.000	.000	.000	.000	.000
	Alpha		.001	.002	.005	.000	.278	.005	.009	.515	.032	.001	.000	.000	.000	.000	.000	.000	.000	.000
	Beta		.076	.000	.041	.014	.022	.000	.000	.045	.000	.000	.000	.000	.000	.000	.000	.000	.000	.000
	Gamma		.009	.000	.016	.014	.050	.000	.000	.011	.000	.000	.000	.000	.000	.000	.000	.000	.000	.000
DE	Delta	Without ELBN	.008	.003	.349	.567	.266	.577	.442	.756	.965	.827	.770	.004	.008	.079	.628	.386	.141	.109
	Theta		.013	.000	.894	.003	.218	.000	.000	.098	.071	.509	.061	.431	.000	.000	.211	.059	.000	.000
	Alpha		.000	.000	.000	.000	.061	.352	.728	.143	.000	.003	.030	.000	.000	.013	.000	.013	.002	.068
	Beta		.000	.001	.056	.476	.494	.000	.002	.000	.011	.000	.000	.114	.000	.013	.063	.466	.000	.007
	Gamma		.202	.243	.000	.000	.007	.126	.005	.000	.001	.018	.120	.000	.054	.000	.000	.001	.086	.000
	Delta	With ELBN	.000	.000	.000	.053	.001	.014	.000	.010	.212	.061	.186	.000	.000	.000	.000	.000	.016	.000
	Theta		.000	.000	.095	.000	.000	.000	.000	.001	.000	.000	.000	.000	.000	.001	.000	.000	.000	.000
	Alpha		.000	.000	.000	.000	.000	.000	.000	.000	.000	.000	.000	.000	.000	.000	.000	.000	.000	.000
	Beta		.000	.000	.000	.000	.000	.000	.000	.000	.000	.000	.000	.000	.000	.000	.000	.000	.000	.000
	Gamma		.000	.000	.000	.000	.000	.000	.000	.000	.000	.000	.000	.000	.000	.000	.000	.000	.000	.000

## Data Availability

Data available in a publicly accessible repository. The data presented in this study are publicly available in SEED dataset at 10.1109/TAMD.2015.2431497, reference number [29].
